# Different clinical characteristics of current smokers and former smokers with asthma: a cross-sectional study of adult asthma patients in China

**DOI:** 10.1038/s41598-022-22953-z

**Published:** 2023-01-19

**Authors:** Zhifeng Chen, Binaya Wasti, Yulin Shang, Ruoyun Ouyang, Yu Yuan, Yi He, Wentao Duan, Jingsi Jia, Bing Xiao, Dongshan Zhang, Shaokun Liu, Qing Song, Yuqin Zeng, Qingping Zeng, Xiufeng Zhang, Jianmin Li, Xiaoying Ji, Ping Chen, Libing Ma, Xudong Xiang

**Affiliations:** 1 Department of Respiratory Medicine, Hunan Centre for Evidence-Based Medicine, Research Unit of Respiratory Diseases, The Second Xiangya Hospital, Central South University, 139 Middle Renmin Road, Changsha, 410011 Hunan China; 2 Ophthalmology and Otorhinolaryngology, Zigui County Traditional Chinese Medicine Hospital, 30 Pinghu Avenue, Zigui, 443600 Hubei China; 3 Department of Emergency, The Second Xiangya Hospital, Central South University, 139 Middle Renmin Road, Changsha, 410011 Hunan China; 4 Department of Respiratory and Critical Care Medicine, Longshan County People’s Hospital, 50 Yuelu Avenue, Longshan, 416800 Hunan China; 5 Department of Respiratory Medicine, The Second Affiliated Hospital of Hainan Medical University, 48 Pak Shui Tong Road, Haikou, 570000 Hainan China; 6 Department of Respiratory and Critical Care Medicine, Hunan Provincial People’s Hospital, 61 West Jiefang Road, Changsha, 410005 Hunan China; 7 Department of Respiratory and Critical Care Medicine, The Affiliated Hospital of Guizhou Medical University, 28 Guiyi Street, Guiyang, 550004 Guizhou China; 8 Department of Respiratory and Critical Care Medicine, The Affiliated Hospital of Guilin Medical University, 15 Le Qun Road, Guilin, 541001 Guangxi China

**Keywords:** Health care, Risk factors

## Abstract

Smoking is a trigger for asthma, which has led to an increase in asthma incidence in China. In smokers, asthma management starts with smoking cessation. Data on predictors of smoking cessation in Chinese patients with asthma are scarce. The objective of this study was to find the differences in clinical characteristics between current smokers and former smokers with asthma in order to identify factors associated with smoking cessation. Eligible adults with diagnosed asthma and smoking from the hospital outpatient clinics (n = 2312) were enrolled and underwent a clinical evaluation, asthma control test (ACT), and pulmonary function test. Information on demographic and sociological data, lung function, laboratory tests, ACT and asthma control questionnaire (ACQ) scores was recorded. Patients were divided into a current smokers group and a former smokers group based on whether they had quit smoking. Logistic regression analysis was used to analyze the factors associated with smoking cessation. Of all patients with asthma, 34.6% were smokers and 65.4% were former smokers, and the mean age was 54.5 ± 11.5 years. Compared with current smokers, the former smokers were older, had longer duration of asthma, had higher ICS dose, had more partially controlled and uncontrolled asthma, had more pack-years, had smoked for longer, and had worse asthma control. The logistic regression model showed that smoking cessation was positively correlated with age, female sex, pack-years, years of smoking, partially controlled asthma, uncontrolled asthma, and body mass index (BMI), but was negatively correlated with ACT, FEV_1_, FEV_1_%predicted, and widowed status. More than 30% of asthma patients in the study were still smoking. Among those who quit smoking, many quit late, often not realizing they need to quit until they have significant breathing difficulties. The related factors of smoking cessation identified in this study indicate that there are still differences between continuing smokers and former smokers, and these factors should be focused on in asthma smoking cessation interventions to improve the prognosis of patients with asthma.

## Introduction

Asthma is a prevalent and highly heterogeneous chronic respiratory inflammatory disease that affects about 300 million people worldwide^1^. Cigarette smoking is one of the preventable triggers of asthma and many previous studies have assessed the relationship between smoking and asthma^2^. Kim et al. found that the incidence of wheezing and exercise-induced wheezing increased with the increase of total pack-years of smoking among current smokers and former smokers^3^. In addition, long-term smoking significantly reduced the sensitivity of asthma patients to inhaled corticosteroids (ICS) and caused a significant decrease in forced expiratory volume in 1 s (FEV_1_)^4,5^.

Recent studies have shown that quitting smoking improves asthma control in smokers with asthma, while airway hyperresponsiveness, neutrophils and fractionated exhaled nitric oxide (FeNO) are reduced, leading to reduced chronic inflammation of the airways^6,7^. Mathias et al. found that smoking cessation was related to the number of years of smoking and the level of education received, and the quit rate of middle-aged smokers was significantly higher^8^. In addition, a new diagnosis of asthma was associated with an increased rate of quitting smoking in a Norwegian study of general trends in smoking cessation^9^. These factors may be related to smoking cessation. However, there is currently little data on smoking cessation among Chinese asthma patients. In this study, we aim to understand the characteristics of asthma in current smokers and former smokers in order to identify the key factors driving smoking cessation.

## Patients and methods

### Study participants and definitions

**Figure 1:**
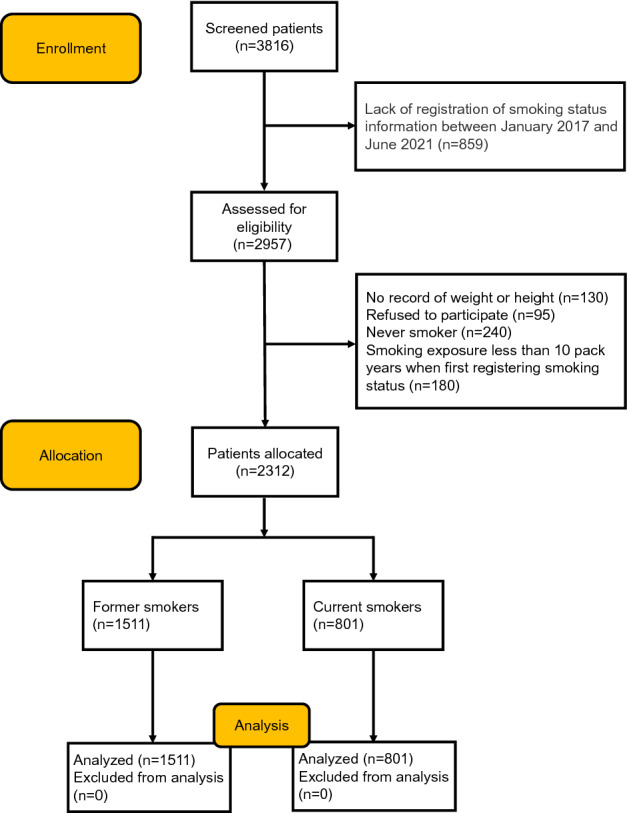
Flow diagram of the study.

This study was approved by the Ethics Review Committee of the Second Xiangya Hospital of Central South University (Ethical Code: LYF2021159), all participants signed an informed consent form and all experiments were performed in accordance with the Declaration of Helsinki. Initially, we included 3816 patients with asthma registered in the outpatient department of the Second Xiangya Hospital of Central South University (Hunan, China) between January 2017 and June 2021. Asthma was diagnosed according to the Global Initiative for Asthma (GINA)^10^, with bronchodilation FEV_1_ change > 200 ml and 12%; Bronchial stimulation test was positive; Symptoms of asthma (including wheezing, difficulty breathing, chest tightness or coughing) occur. All patients were treated with a combination of ICS and long-acting β_2_ agonist (ICS/LABA). The non-smokers were defined as participants who had never smoked or had smoked fewer than 100 cigarettes in their lifetime. Smokers were defined as having smoked continuously for more than 10 pack-years. The former smokers were defined as participants who had quit smoking for at least 6 months prior to the study. Patients who had never smoked, had no registered smoking history, had less than 10 pack-years, and were younger than 18 years old were excluded. A detailed description of the flow diagram for recruiting voluntary patients can be found in Fig. 1.

### Data collection

After the collection of written informed consent, participants’ age, sex, duration of asthma, drug treatment, exacerbation, asthma and chronic obstructive pulmonary disease overlap (ACO) occurrence, education level, marital status and smoking status were documented. Height and weight were measured, and body mass index (BMI) was calculated. Meanwhile, laboratory tests [e.g., immunoglobulin E (IgE), blood eosinophils, blood neutrophils, FeNO], asthma control test (ACT), asthma control questionnaire (ACQ), asthma control, and pulmonary function test (PFT) results were recorded. This included FEV_1_ and forced vital capacity (FVC), and the values of FEV_1_/FVC and FEV_1_%predicted were calculated. ACT scores range from 0 to 25, with a score of 20–25 indicating good asthma control and a score below 20 indicating poor asthma control^11^. ACQ consists of seven items, each of which ranges from 0 (fully controlled) to 6 (severely uncontrolled). ACQ scores are the average of the seven items and current studies have established the cut-off values for controlled asthma (ACQ ≤ 0.75 points) and poorly controlled asthma (ACQ ≥ 1.5 points)^12^.

### Patient selection

A total of 2312 patients (including 1511 former smokers and 801 current smokers) were included in this study, excluding 1504 patients (859 patients with no smoking history, 130 patients with no record of weight or height, 95 patients who refused to participate, 180 patients who smoked less than 10 pack-years, and 240 never-smokers).

### Statistical analysis

SPSS 26.0 software (IBM Corp.) was used to perform all statistical analyses, and GraphPad Prism 8.0.1 software (GraphPad Software Inc) was used to generate the graphs. Continuous variables were described as the mean and standard deviation (SD) or median (interquartile range, IQR), and categorical variables were expressed as the number (percentage). Differences between the two groups were determined using Student’s *t* test, and the Mann–Whitney *U* test. Multivariate logistic regression was used to calculate the odds ratio (ORs) of various adjustments. A *P*-value < 0.05 indicated a statistically significant difference.

### Statement of ethics

This study was approved by the Ethics Review Committee of the Second Xiangya Hospital of Central South University (Ethical Code: LYF2021159), all participants signed an informed consent form and all experiments were performed in accordance with the Declaration of Helsinki.

## Results

### Demographic characteristics

Table 1 shows the demographic and sociological characteristics, lung function indexes and biochemical indexes of the 2312 participants, including 801 current smokers and 1511 former smokers. The mean age was 52.4 ± 11.2 years for current smokers and 55.5 ± 11.1 years for former smokers (Table 1). There were significant differences in age, sex, asthma duration, marital status, education and BMI between the current smokers and former smokers. Compared with the current smokers, the former smokers were older, had longer duration of asthma, had higher ICS dose, had more partially controlled and uncontrolled asthma, had lower ACT scores, FEV_1_, FEV_1_/FVC, and FEV_1_%predicted, as well as more pack-years and longer duration of smoking. Interestingly, the current smokers had higher IgE, FeNO, blood eosinophils, and blood neutrophils, but lower mean ACQ scores compared to the former smokers. Detailed information regarding participant characteristics is shown in Table 1.

**Table 1:** Clinical characteristics of participants.

Items	Total	Current smokers	Former smokers	*P* value
Subjects, n (%)	2312	801 (34.6)	1511 (65.4)	
**Age (years), M ± SD**	54.5 ± 11.3	52.4 ± 11.2	55.5 ± 11.1	< 0.001
< 45, n (%)	396 (17.1)	171 (21.3)	225 (14.9)	< 0.001
45–64	1428 (61.8)	535 (66.8)	893 (59.1)	
≥ 65	488 (21.1)	95 (11.9)	393 (26.0)	
**Sex, n (%)**	2312	801 (34.6)	1511 (65.4)	< 0.001
Male	1233 (53.3)	536 (66.9)	697 (46.1)	
Female	1079 (46.7)	265 (33.1)	814 (53.9)	
Duration of asthma (years), M ± SD	17.9 ± 10.5	15.7 ± 9.3	19.1 ± 11.0	< 0.001
**Marriage, n (%)**	2312	801 (34.6)	1511 (65.4)	< 0.001
Married	1927 (83.3)	621 (77.5)	1306 (86.4)	
Unmarried	166 (7.2)	144 (18.0)	22 (1.5)	
Widow	219 (9.5)	36 (4.5)	183 (12.1)	
BMI (kg/m^2^), M ± SD	22.9 ± 2.4	22.4 ± 2.2	23.2 ± 2.5	< 0.001
**Education, n (%)**	2312	801 (34.6)	1511 (65.4)	< 0.001
Primary school	581 (25.1)	125 (15.6)	456 (30.2)	
High school	1294 (56.0)	451 (56.3)	843 (55.8)	
University	437 (18.9)	225 (28.1)	212 (14.0)	
Pack-years, M ± SD	18.5 ± 8.9	15.2 ± 7.4	20.2 ± 9.1	< 0.001
**Amount smoked, n (%)**	2312	801 (34.6)	1511 (65.4)	0.006
< 2 packs/day	1795 (77.6)	593 (74.0)	1202 (79.5)	
≥ 2 packs/day	517 (22.4)	208 (26.0)	309 (20.5)	
**Years of smoking (years)**	14.7 ± 6.6	12.4 ± 7.1	15.9 ± 6.0	0.011
ACT, M ± SD	21.5 ± 3.5	22.5 ± 3.1	20.9 ± 3.5	< 0.001
ACQ, M ± SD	0.65 ± 0.38	0.61 ± 0.32	0.68 ± 0.40	< 0.001
**Asthma control, n (%)**	2312	801 (34.6)	1511 (65.4)	< 0.001
Well controlled	1849 (80.0)	710 (88.6)	1139 (75.4)	
Partially controlled	393 (17.0)	73 (9.1)	320 (21.2)	
Uncontrolled	70 (3.0)	18 (2.2)	52 (3.4)	
**Lung function indexes, median (IQR)**				
FEV_1_ (L)	2.34 (1.84–2.93)	2.46(1.92–3.32)	2.30 (1.57–2.73)	0.010
FEV_1_%predicted (%)	76.8 (56.7–92.5)	84.7 (65.5–95.6)	73.4 (49.0–89.4)	0.006
FEV_1_/FVC (%)	70.3 (60.5–76.2)	71.2 (63.3–78.4)	70.2 (57.4–75.3)	0.013
ICS dose at entry, μg/day, M ± SD	201.3 ± 92.6	182.0 ± 62.6	211.5 ± 103.6	< 0.001
Rate of exacerbation in past 12 months, n (%)	152 (6.6)	47 (5.9)	105 (6.9)	0.333
ACO, n (%)	304 (13.1)	95 (11.9)	209 (13.8)	0.196
**Biochemical indexes, median (IQR)**				
IgE (mg/l)	257.8 (105.7–430.6)	391.2 (228.1–710.3)	216.6 (78.4–333.1)	0.012
FeNO (ppb)	65.0 (35.0–130.0)	86.0 (43.0–137.0)	58.0 (32.0–126.0)	< 0.001
Blood eosinophils (× 10^9^)	0.42 (0.22–0.64)	0.47 (0.31–0.66)	0.39 (0.20–0.62)	0.018
Blood neutrophils (× 10^9^)	3.48 (3.01–4.35)	4.47 (4.15–5.96)	3.19 (2.64–3.56)	0.009

Comparisons were determined using Student’s *t* test and the Mann–Whitney *U* test between two groups, and the chi-square test, one-way analysis of variance (ANOVA) or the Kruskal–Wallis test followed by Dunn’s multiple comparisons test between three groups. *P* < 0.05 was considered statistically significant.

*ACQ* asthma control questionnaire, *ACO* asthma and chronic obstructive pulmonary disease overlap, *ACT* asthma control test, *BMI* body mass index, *FeNO* fractionated exhaled nitric oxide, *FEV*_*1*_ forced expiratory volume in 1 s, *FVC* forced vital capacity, *ICS* inhaled corticosteroids, *IgE* immunoglobulin E, *IQR* interquartile range, *LABA* long-acting β_2_ agonist, *M ± SD* mean ± standard deviation, *ppb* parts per billion.

### Multivariate logistic regression analysis of factors associated with smoking cessation based on clinical parameters and sociodemographic characteristics

Multivariate logistic regression was used to analyze the factors significantly associated with smoking cessation based on clinical parameters and sociodemographic characteristics. According to the clinical parameters, ACT was negatively associated with smoking cessation, with an OR of 0.907 (95% CI = 0.862–0.956), while partially controlled and uncontrolled asthma were positively correlated with smoking cessation, with an OR of 2.733 and 1.801 (95% CI = 2.084–3.583, 1.045–3.103), respectively. Patients were more likely to quit smoking if they had poor pulmonary function, with an OR of 0.722 (95% CI = 0.629–0.828) for FEV_1_ and 0.991 for FEV_1_%predicted (95% CI = 0.986–0.996) (Table 2). According to the sociodemographic characteristics, patients who were widowed were less likely to quit, with an OR of 0.059 (95% CI = 0.025–0.142). BMI was positively correlated with smoking cessation, with an OR of 1.156 (95% CI = 1.114–1.199). Patients who were older and had more pack-years were more likely to quit smoking, with an OR of 2.789 and 1.063 (95% CI = 1.008–7.716, 1.039–1.088), respectively. Patients who had longer asthma duration and higher ICS dose were more likely to quit smoking, with an OR of 1.023 and 1.441 (95% CI = 1.013–1.033, 1.053–1.972), respectively. Patients who had more years of smoking were more likely to quit smoking, with an OR of 1.033 (95% CI = 1.002–1.064). Meanwhile, females were also more likely to quit smoking compared to males, with an OR of 3.694 (95% CI = 2.770–4.926) (Table 3).

**Table 2:** Multivariate logistic regression analysis of factors associated with smoking cessation based on clinical parameters.

Variable	OR	95% CI	*P* value
ACT	0.907	0.862–0.956	< 0.001
**Asthma control**			
Well controlled	Reference		
Partially controlled	2.733	2.084–3.583	< 0.001
Uncontrolled	1.801	1.045–3.103	0.034
FEV_1_	0.722	0.629–0.828	< 0.001
FEV_1_%predicted	0.991	0.986–0.996	0.001

Values were expressed as odds ratio (OR) and 95% confidence interval (CI). Factors associated with smoking cessation were determined by multivariate logistic regression analysis. Multivariate analysis was adjusted for ACT, FEV_1_, FEV_1_/FVC, age, sex, marriage, BMI, Pack-years and Years of smoking. *P* < 0.05 was considered statistically significant.

*ACT* asthma control test, *95%CI* 95% confidence interval, *FEV*_*1*_ forced expiratory volume in 1 s, *FVC* forced vital capacity, *OR* odds ratio.

**Table 3:** Multivariate logistic regression analysis of factors associated with smoking cessation based on sociodemographic characteristics.

Variable	OR	95% CI	*P* value
**Age**			
< 45	Reference		
45–64	0.660	0.341–1.278	0.218
≥ 65	2.789	1.008–7.716	0.048
**Sex**			
Male	Reference		
Female	3.694	2.770–4.926	< 0.001
Duration of asthma	1.023	1.013–1.033	< 0.001
**Marriage**			
Married	Reference		
Unmarried	0.942	0.554–1.601	0.825
Widowed	0.059	0.025–0.142	< 0.001
BMI	1.156	1.114–1.199	< 0.001
Pack-years	1.063	1.039–1.088	< 0.001
Years of smoking	1.033	1.002–1.064	0.034
ICS dose	1.441	1.053–1.972	0.022

Values were expressed as odds ratio (OR) and 95% confidence interval (CI). Factors associated with smoking cessation were determined by multivariate logistic regression analysis. Multivariate analysis was adjusted for ACT, FEV_1_, FEV_1_/FVC, age, sex, marriage, BMI, Pack-years and Years of smoking. *P* < 0.05 was considered statistically significant.

*BMI* body mass index, *95% CI* 95% confidence interval, *ICS* inhaled corticosteroids, *OR* odds ratio.

## Discussion

Asthma, characterized by chronic airway inflammation, is a highly heterogeneous disease. This cross-sectional descriptive study including outpatients with asthma compares the different characteristics of current smokers and former smokers. Previous studies have not found any relationship between smoking and asthma control^13^. However, in this study, we found that compared with the current smokers, the former smokers were older, had longer duration of asthma, had higher ICS dose, had lower ACT scores, FEV_1_, FEV_1_/FVC, FEV_1_%predicted and more pronounced asthma symptoms. These findings suggest that most asthma patients continue to smoke until they experience significant symptoms of wheezing, discomfort and shortness of breath. Several studies have found that smoking contributes to increased morbidity and mortality, exacerbation of symptoms and frequent hospitalizations in asthmatic patients^14–16^. At the same time, asthmatics in the smoking group had more frequent asthma attacks, an increased number of life-threatening asthma attacks, and a higher mortality rate among heavy smokers compared to asthmatic non-smokers^17–19^. Polosa et al. found that duration of smoking and smoking status were significantly associated with asthma severity in a dose-dependent manner, with the most significant association with disease severity observed among smokers who smoked for more than 20 pack-years^20^. Evidence of causality is supported by a significant association between asthma severity and active smoking and a clear dose–response relationship. Smoking can cause acute constriction of the bronchi in patients with asthma, resulting in reduced lung function, and the effect of smoking on reduced lung function in patients with chronic obstructive lung disease has been proven^21^. In addition, long-term exposure to cigarette smoke can promote proliferation and activation of bronchial epithelial cells, goblet cells, smooth muscle cells and fibroblasts, leading to excessive secretion of mucus, fibrosis, extracellular matrix deposition and airway remodeling, leading to accelerated decline of FEV_1_ and increased severity of airflow obstruction^21,22^.

Studies have shown that asthmatic smokers who quit smoking have significantly improved quality of life, and reduced nighttime and daytime rescue β2-agonist use, ICS use, daytime asthma symptoms and airway hyperreactivity. They also show increased sensitivity to ICS, and improved asthma management^21,23^. Therefore, the daily management of asthma patients should strengthen the propaganda and education of smoking cessation, in order to improve symptoms and prevent the deterioration of the condition. The current study found some factors associated with smoking cessation. We observed a significant negative association between ACT and smoking cessation. Patients with well-controlled asthma are less likely to quit smoking. We found that FEV_1_ and FEV_1_%predicted were negatively associated with smoking cessation, and patients were more likely to quit smoking when they had dyspnea and worsening symptoms. Our results are consistent with the findings of Godtfredsen et al. that smoking cessation is promoted when lung function is impaired^24^. We found that duration of asthma and ICS dose were positively associated with smoking cessation. Studies have shown that long-term smoking can promote the occurrence of fixed airflow obstruction and induce ICS resistance to increase ICS dose^25–27^. We observed a positive correlation between age and smoking cessation, with smokers becoming more aware of the need to quit as they get older, consistent with previous studies^18^. A study has found that quitting behavior varies by age group, with those over 50 more likely to quit^28^. We found that widowed patients were less likely to quit smoking than married patients, probably because they lived alone. Studies have shown that people who live with a partner or who are married are more likely to quit, those who live alone are less likely to quit, and quitting is more likely to fail if their partner is also a smoker^29–31^. We observed that females are more likely to quit smoking than males, consistent with previous research^32^. In this study, we found that compared with the current smokers, the former smokers had higher BMI, which is consistent with previous findings and may be related to increased appetite after quitting^33,34^. We also found a positive association between BMI and smoking cessation. Studies have shown that BMI has been identified as a risk factor for the development of asthma, the incidence of asthma increases with obesity, and obese individuals are more difficult to control, which is more likely to promote smoking cessation^35–37^. However, it may reduce the likelihood of quitting, which can be accompanied by weight gain^33^. We discovered that patients who had consumed more pack-years and had more years of smoking were more likely to quit smoking. Studies have shown that health scares can influence the smoking behavior of smokers, including those of family or friends. Wang et al. found that health scares reduced the likelihood of heavy smoking (> 20 cigarettes/day) by 41.6% compared with moderate and light smoking, and increased the likelihood of ever smokers to quit by 85.3%^38^. The duration and total pack-years of smoking were positively correlated with wheezing and exercise wheezing^3^. Meanwhile, studies showed that the amount of smoking is an important factor in successful quitting, and smokers who consumed more cigarettes were more likely to quit^32,39^.

There were some limitations of this study: (a) This is a cross-sectional descriptive study; therefore, we cannot draw conclusions about the direction of causation, and the results of this study can only provide data related to smoking cessation, but not on predictive factors. (b) Since we relied on self-reports to determine smoking status, patients’ desire to respond to social expectations might lead to underestimation of smoking status, as well as recall bias. (d) The mechanisms of the factors associated with smoking cessation are unexplained and need to be further explored.

## Conclusions

In conclusion, more than 30% of asthma patients in the study were still smoking. Among those who quit smoking, many quit late, often not realizing they need to quit until they have significant breathing difficulties. The related factors of smoking cessation identified in this study indicate that there are still differences between continuing smokers and former smokers, and these factors should be focused on in asthma smoking cessation interventions to improve the prognosis of patients with asthma (Supplementary information).

## Supplementary Information

Supplementary Information 1.

Supplementary Information 2.

## Data Availability

The data used and analyzed in this study are available from the corresponding author on reasonable request; E-mail: xudongxiang@csu.edu.cn.

## Abbreviations

ACQ: Asthma control questionnaire
ACT: Asthma control test
BMI: Body mass index
CI: Confidence interval
FeNO: Fractionated exhaled nitric oxide
FEV1: Forced expiratory volume in 1 s
FVC: Forced vital capacity
HAT: Histone acetylases
HDAC: Histone deacetylases
ICS: Inhaled corticosteroids
IgE: Immunoglobulin E
IL-4: Interleukin-4
IQR: Interquartile range
OR: Odd ratio
PFT: Pulmonary function test
Ppb: Parts per billion
M ± SD: Mean ± standard deviation
T2: Type 2
Th2: T-helper cell type 2

## References

[1] Zhang F, Hang J, Zheng B, Su L, Christiani DC (2015). The changing epidemiology of asthma in Shanghai, China. J. Asthma.

[2] Beasley R, Semprini A, Mitchell EA (2015). Risk factors for asthma: is prevention possible?. Lancet.

[3] Kim SY, Sim S, Choi HG (2018). Active and passive smoking impacts on asthma with quantitative and temporal relations: A Korean Community Health Survey. Sci. Rep..

[4] Thomson NC, Chaudhuri R (2009). Asthma in smokers: Challenges and opportunities. Curr. Opin. Pulm. Med..

[5] Shimoda T, Obase Y, Kishikawa R, Iwanaga T (2016). Influence of cigarette smoking on airway inflammation and inhaled corticosteroid treatment in patients with asthma. Allergy Asthma Proc..

[6] Westergaard CG, Porsbjerg C, Backer V (2014). The effect of smoking cessation on airway inflammation in young asthma patients. Clin. Exp. Allergy.

[7] Piccillo G (2008). Changes in airway hyperresponsiveness following smoking cessation: Comparisons between Mch and AMP. Respir. Med..

[8] Holm M (2017). Predictors of smoking cessation: A longitudinal study in a large cohort of smokers. Respir. Med..

[9] Danielsen SE, Løchen ML, Medbø A, Vold ML, Melbye H (2016). A new diagnosis of asthma or COPD is linked to smoking cessation—The Tromsø study. Int. J. Chron. Obstruct. Pulmon. Dis..

[10] Boulet, L. P. *et al.* The Global Initiative for Asthma (GINA): 25 years later. *Eur. Respir. J*. 10.1183/13993003.00598-2019 (2019).

[11] Nathan RA (2004). Development of the asthma control test: A survey for assessing asthma control. J. Allergy Clin. Immunol..

[12] Jia CE (2013). The asthma control test and asthma control questionnaire for assessing asthma control: Systematic review and meta-analysis. J. Allergy Clin. Immunol..

[13] González Barcala, F. J. *et al.* Factors associated with asthma control in primary care patients: The CHAS study. *Arch. Bronconeumol.* **46**, 358–363. 10.1016/j.arbres.2010.01.007 (2010). 10.1016/j.arbres.2010.01.007 20227808

[14] Santos V (2022). Association of quality of life and disease control with cigarette smoking in patients with severe asthma. Braz. J. Med. Biol. Res..

[15] Hough KP (2020). Airway remodeling in asthma. Front. Med. (Lausanne).

[16] Shavit O (2007). Impact of smoking on asthma symptoms, healthcare resource use, and quality of life outcomes in adults with persistent asthma. Qual. Life Res..

[17] Gonzalez-Barcala FJ (2018). Asthma exacerbations: Risk factors for hospital readmissions. Ir. J. Med. Sci..

[18] Blakey JD (2017). Identifying risk of future asthma attacks using UK medical record data: A respiratory effectiveness group initiative. J. Allergy Clin. Immunol. Pract..

[19] Patel SN (2009). Multicenter study of cigarette smoking among patients presenting to the emergency department with acute asthma. Ann. Allergy Asthma Immunol..

[20] Polosa R (2011). Greater severity of new onset asthma in allergic subjects who smoke: A 10-year longitudinal study. Respir. Res..

[21] Polosa R, Thomson NC (2013). Smoking and asthma: Dangerous liaisons. Eur. Respir. J..

[22] Wilson SJ (2021). Airway elastin is increased in severe asthma and relates to proximal wall area: Histological and computed tomography findings from the U-BIOPRED severe asthma study. Clin. Exp. Allergy.

[23] Tønnesen P (2005). Effects of smoking cessation and reduction in asthmatics. Nicotine Tob. Res..

[24] Huang K (2019). Prevalence, risk factors, and management of asthma in China: A national cross-sectional study. Lancet.

[25] Bennett GH (2018). Risk factors and clinical outcomes associated with fixed airflow obstruction in older adults with asthma. Ann. Allergy Asthma Immunol..

[26] Jabbal S, Kuo CR, Lipworth B (2020). Randomized controlled trial of triple versus dual inhaler therapy on small airways in smoking asthmatics. Clin. Exp. Allergy.

[27] Graff S (2020). Clinical and biological factors associated with irreversible airway obstruction in adult asthma. Respir. Med..

[28] Chen D, Wu LT (2015). Smoking cessation interventions for adults aged 50 or older: A systematic review and meta-analysis. Drug Alcohol Depend..

[29] Tøttenborg SS, Thomsen RW, Johnsen SP, Nielsen H, Lange P (2016). Determinants of smoking cessation in patients with COPD treated in the outpatient setting. Chest.

[30] Lee CW, Kahende J (2007). Factors associated with successful smoking cessation in the United States, 2000. Am. J. Public Health.

[31] Lou P (2013). Supporting smoking cessation in chronic obstructive pulmonary disease with behavioral intervention: A randomized controlled trial. BMC Fam. Pract..

[32] Kim YJ (2014). Predictors for successful smoking cessation in Korean adults. Asian Nurs. Res. (Korean Soc. Nurs. Sci.).

[33] Ben Taleb, Z. *et al.* Smoking cessation and changes in body mass index: Findings from the first randomized cessation trial in a low-income country setting. *Nicotine Tob. Res.* **19**, 351–356. 10.1093/ntr/ntw223 (2017). 10.1093/ntr/ntw223 PMC13648115 27613912

[34] Tian J, Venn A, Otahal P, Gall S (2015). The association between quitting smoking and weight gain: A systematic review and meta-analysis of prospective cohort studies. Obes. Rev..

[35] Brumpton B, Langhammer A, Romundstad P, Chen Y, Mai XM (2013). General and abdominal obesity and incident asthma in adults: The HUNT study. Eur. Respir. J..

[36] Boudreau M, Bacon SL, Ouellet K, Jacob A, Lavoie KL (2014). Mediator effect of depressive symptoms on the association between BMI and asthma control in adults. Chest.

[37] van Zelst CM (2021). Association between elevated serum triglycerides and asthma in patients with obesity: An explorative study. Allergy Asthma Proc..

[38] Wang, Q., Rizzo, J. A. & Fang, H. Changes in Smoking Behaviors following Exposure to Health Shocks in China. *Int. J. Environ. Res. Public Health*. 10.3390/ijerph15122905 (2018). 10.3390/ijerph15122905 PMC6313584 30572581

[39] Godtfredsen NS, Prescott E, Osler M, Vestbo J (2001). Predictors of smoking reduction and cessation in a cohort of danish moderate and heavy smokers. Prev. Med..

