# Supplementary material for: Different clinical characteristics of current smokers and former smokers with asthma: a cross-sectional study of adult asthma patients in China
Source: Sci Rep. 2023 Jan 19;13:1035. doi: 10.1038/s41598-022-22953-z (PMC9852572; doi:10.1038/s41598-022-22953-z)
Supplement: Supplementary file 1 — Supplementary Information 1. [file 41598_2022_22953_MOESM1_ESM.docx]

The Asthma Control Questionnaire

1. On average, during the past week, how often were you woken by your asthma during the night? Never (0); Hardly ever (1); A few times (2); Several times (3); Many times (4); A great many times (5); Unable to sleep because of asthma (6 points).

2. On average, during the past week, how bad were your asthma symptoms when you woke up in the morning? No symptoms (0); Very mild symptoms (1); Mild symptoms (2); Moderate symptoms (3); Quite severe symptoms (4); Severe symptoms (5); Very severe symptoms (6).

3. In general, during the past week, how limited were you in your activities because of your asthma? Not limited at all (0); Very slightly limited (1); Slightly limited (2); Moderately limited (3); Very limited (4); Extremely limited (5); Totally limited (6).

4. In general, during the past week, how much shortness of breath did you experience because of your asthma? None (0); A very little (1); A little (2); A moderate amount (3); Quite a lot (4); A great deal (5); A very great deal (6).

5. In general, during the past week, how much of the time did you wheeze? Never (0); Hardly any of the time (1); A little of the time (2); A moderate amount of the time (3); A lot of the time (4); Most of the time (5); All the time (6).

6. On average, during the past week, how many puffs/inhalations of short-acting bronchodilator? (eg, Ventolin, Bricanyl) have you used each day? (If you are not sure how to answer this question, please ask for help.) None (0); 1–2 puffs/inhalations most days (1); 3–4 puffs/inhalations most days (2); 5–8 puffs/inhalations most days (3); 9–12 puffs/inhalations most days (4); 13–16 puffs/inhalations most days (5); More than 16 puffs/inhalations most days (6).

7. FEV_1_% predicted before bronchodilator. >95% predicted (0); 95%-90% (1); 89%-80% (2); 79%-70% (3); 69%-60% (4); 59%-50% (5); <50% predicted (6).

Total scores are the mean of the seven items:
