# Supplementary material for: Different clinical characteristics of current smokers and former smokers with asthma: a cross-sectional study of adult asthma patients in China
Source: Sci Rep. 2023 Jan 19;13:1035. doi: 10.1038/s41598-022-22953-z (PMC9852572; doi:10.1038/s41598-022-22953-z)
Supplement: Supplementary file 2 — Supplementary Information 2. [file 41598_2022_22953_MOESM2_ESM.docx]

The Asthma Control Test

1. In the past 4 weeks, how much of the time did your asthma keep you from getting as much done at work, school or at home? All of the time (1 point); Most of the time (2); Some of the time (3); A little of the time (4); None of the time (5 points).

2. During the past 4 weeks, how often have you had shortness of breath? More than once a day (1); Once a day (2); 3 to 6 times a week (3); Once or twice a week (4); Not at all (5).

3. During the past 4 weeks, how often did your asthma symptoms (wheezing, coughing, shortness of breath, chest tightness or pain) wake you up at night or earlier than usual in the morning? 4 or more nights a week (1); 2 to 3 nights a week (2); Once a week (3); Once or twice (4); Not at all (5).

4. During the past 4 weeks, how often have you used your rescue inhaler or nebulizer medication (such as salbutamol)? 3 or more times per day (1); 1 or 2 times per day (2); 2 or 3 times per week (3); Once a week or less (4); Not at all (5).

5. How would you rate your asthma control during the past 4 weeks? Not controlled at all (1); Poorly controlled (2); Somewhat controlled (3); Well controlled (4); Completely controlled (5).

Total points:
